# Beneficial effect of KYP-2047, a propyl-oligopeptidase inhibitor, on oral squamous cell carcinoma

**DOI:** 10.18632/oncotarget.28147

**Published:** 2021-12-07

**Authors:** Sarah Adriana Scuderi, Giovanna Casili, Alessia Filippone, Marika Lanza, Rossella Basilotta, Raffaella Giuffrida, Stefania Munaò, Lorenzo Colarossi, Anna Paola Capra, Emanuela Esposito, Irene Paterniti

**Affiliations:** ^1^Department of Chemical, Biological, Pharmaceutical and Environmental Sciences, University of Messina, Viale Ferdinando Stagno D’ Alcontres, Messina 31-98166, ME, Italy; ^2^IOM Ricerca Srl, Viagrande 95029, CT, Italy; ^3^Istituto Oncologico del Mediterraneo, Viagrande 95029, CT, Italy; ^4^Department of Clinical and Experimental Medicine, University of Messina, Viale Ferdinando Stagno D’ Alcontres, Messina 31-98166, ME, Italy; ^*^These authors contributed equally to this work

**Keywords:** oral squamous cell carcinoma (OSCC), tongue squamous cell carcinoma (TSCC), prolyl-oligopeptidase (POP), angiogenesis, apoptosis

## Abstract

Oral squamous cell-carcinoma (OSCC) is a common cancer which arises from the alveolar ridge, buccal mucosa, and tongue. Among OSCC, the incidence of tongue squamous cell-carcinoma (TSCC) is growing all over the world. Oral carcinogenesis has been linked to genetic mutations, chromosomal aberrations and viral factors. Apoptosis and angiogenesis play a key role in the development of oral cancer. Therefore, it is very important discover new therapeutic strategies to counteract oral cancer progression. This study aimed to investigate the effect of KYP-2047 in an *in vitro* model of TSCC and *in vivo* CAL27-xenograft model. Our results demonstrated that KYP-2047 was able to reduce TSCCs cell viability at the concentrations of 50 μM and 100 μM. Additionally, KYP-2047 was able to increase Bax, Bad and caspase-3 expression, whereas Bcl-2 and p53 expression were reduced. Moreover, KYP-2047 significantly reduced vascular-endothelial-growth-factor (VEGF) and endothelial-nitric-oxide-synthase (eNOS) expression. In the *vivo* xenograft model, KYP-2047 at doses of 1 and 5 mg/kg significantly reduced tumor burden and tumor weight, decreasing also angiogenesis markers VEGF and eNOS. Moreover, KYP-2047 increased Bax and reduced Bcl2 expressions. Thus, KYP-2047 could represent a potential therapeutic treatment to counteract tongue oral-cancer growth, thanks its abilities to modulate angiogenesis and apoptosis pathways.

## INTRODUCTION

Oral squamous cell carcinoma (OSCC) is a cancer which arises from mucosal lining of the oral cavity with strong invasion and metastasis ability [1–3]. It is estimated that there were almost 350.000 new OSCC cases per year with poor prognosis [2, 3]. Tongue squamous cell carcinoma (TSCC) is a common cancer of oral cavity; its incidence increased worldwide [4]. The progression of TSCC is related often to various risk factors as smoking and excessive alcohol consumption [5, 6], and genetic factors such as chromosomal aberrations, tumor suppressor genes, oncogenes, and DNA mismatch repair genes [7, 8]. Currently, therapy for locally oral cancer treatment includes surgical resection, chemotherapy and postoperative radiation [1]. However, the survival percentage of patients affected by oral cancer remains very low, therefore the research of new approaches therapeutics is needed to improve patient outcomes [1].

Scientific evidences demonstrated that apoptosis and angiogenesis processes contribute to the development of oral cancer [2]. Apoptosis is a process of cell death that arises when DNA injury is irreversible [9]. Studies have shown that alterations of key regulatory apoptosis factors may lead to cancer, promoting metastasis and resistance to chemotherapy drugs [9, 10]. On the other hand, also angiogenesis contributes to the development of oral cancer [11]. Angiogenesis is a complex phenomenon that is essential for the growth and progression of solid neoplasms as oral cancer [10, 11]. Mutations or further alterations consent to cancer cells to proliferate excessively, and to migrate to the basal membrane, promoting angiogenesis [12, 13]. Therefore, it is very important discover new therapeutic strategies able to counteract or reduce oral cancer progression. In this context, more attention was given to the role of prolyl-oligopeptidase (POP) in cancer. Prolyl-oligopeptidase (POP) or propyl endopeptidase (PREP), is a proteinase constitutively expressed in all cells [14–16]. POP enzyme modulates proliferation, the formation of new blood vessels and cell death processes which can contribute to cancer pathogenesis [16]. Recent studies have proven the involvement of POP enzyme in cancer progression, in particular in glioblastoma, breast and gastric cancer, suggesting the development of potential POP-inhibitors as a promising strategy for cancer treatment [17–19].

Considering the fundamental role of POP in cancer pathogenesis, in the last decade several POP-inhibitors, as KYP-2047, have been developed to investigate their beneficial effect in many pathologies including cancer, showing promising results [15, 20, 21]. Therefore, this paper aimed to investigate the ability of KYP-2047 to modulate angiogenesis and apoptosis pathways in an *in vitro* model of TSCC on CAL27, HSC-2 and HSC-3 cell cultures and in an *in vivo* CAL27-xenograft model.

## RESULTS

### *In vitro* studies

#### KYP-2047 reduced CAL27, HSC-2 and HSC-3 cell viability

**Figure 1:**
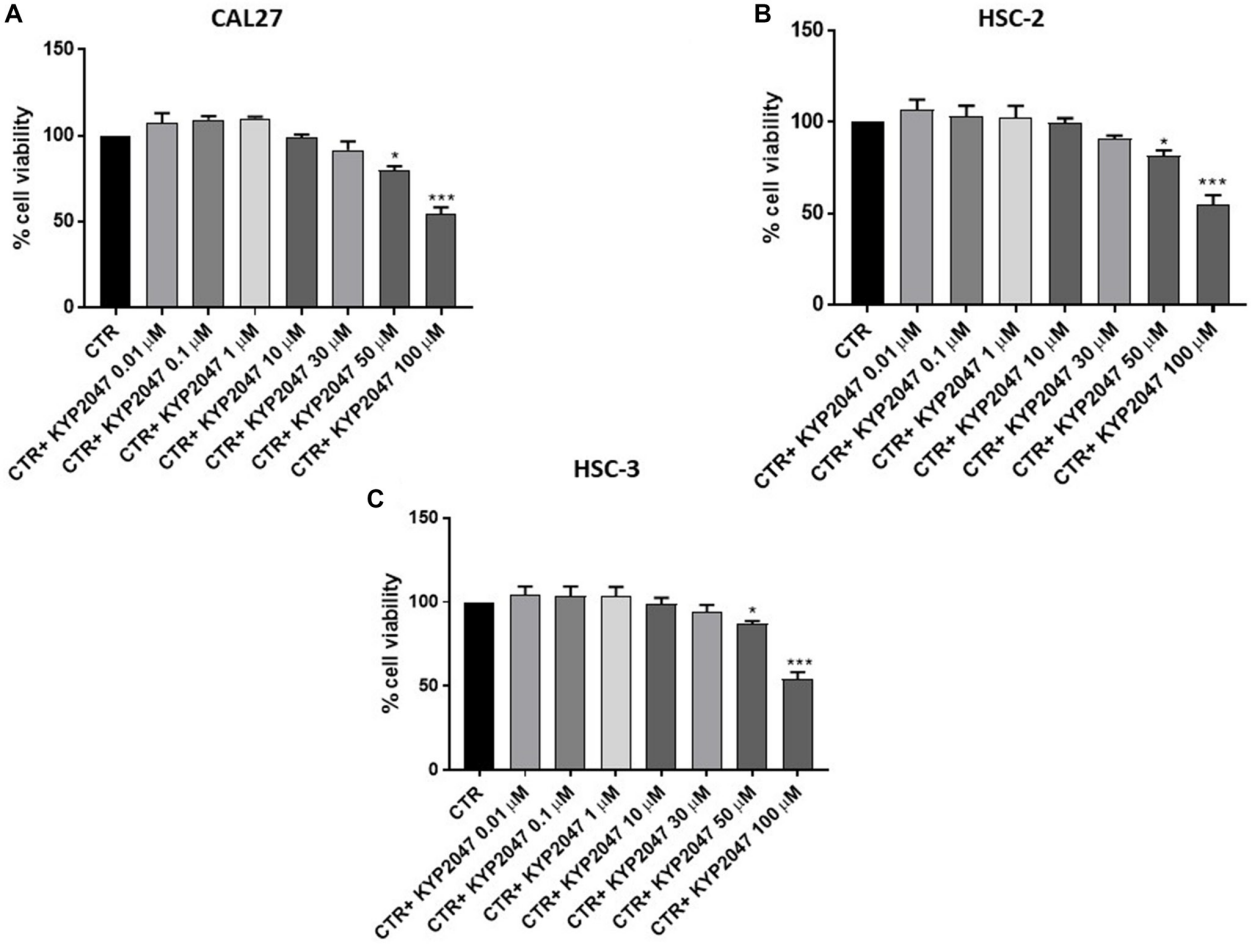
Effect of KYP-2047 on CAL27, HSC-2 and HSC-3 cell viability. KYP-2047 treatment only at concentrations of 50 μM and 100 μM reduced significantly cell viability in all three cell lines. Data are representative of at least three independent experiments. (**A**) ^*^ *p* < 0.05 vs. CTR; ^***^ *p* < 0.001 vs. CTR. (**B**) ^*^ *p* < 0.05 vs. CTR; ^***^ *p* < 0.001 vs. CTR. (**C**) ^*^ *p* < 0.05 vs. CTR; ^***^ *p* < 0.001 vs. CTR.

CAL27, HSC-2 and HSC-3 cell viability was assessed following 24 hrs of treatment with KYP-2047 at different concentrations. The treatment with KYP-2047 only at higher concentrations was able to reduce significantly viability in all cell lines as shown in Figure 1A–1C.

Since KYP-2047 showed similar effects on cell viability in all cell cultures, we decided to continue to analyze the effect of KYP-2047 only on CAL27 cell line because represented one of the most frequently used cell line in the field of OSCC and validated from neck and head tumor sites [22–24].

#### KYP-2047 increased apoptosis in CAL27 cells

Apoptosis is an important cellular process that occurs in physiological and pathological conditions as cancer [25, 26]. Alterations of apoptosis pathway can lead to malignant transformation of cells, tumour progression, metastasis and resistance to chemotherapy drugs [25, 26]. Therefore, in this paper we detected the effect of KYP-2047 on apoptosis pathway by evaluating apoptotic markers as Bax, Bad, Caspase 3 and Bcl2.

**Figure 2:**
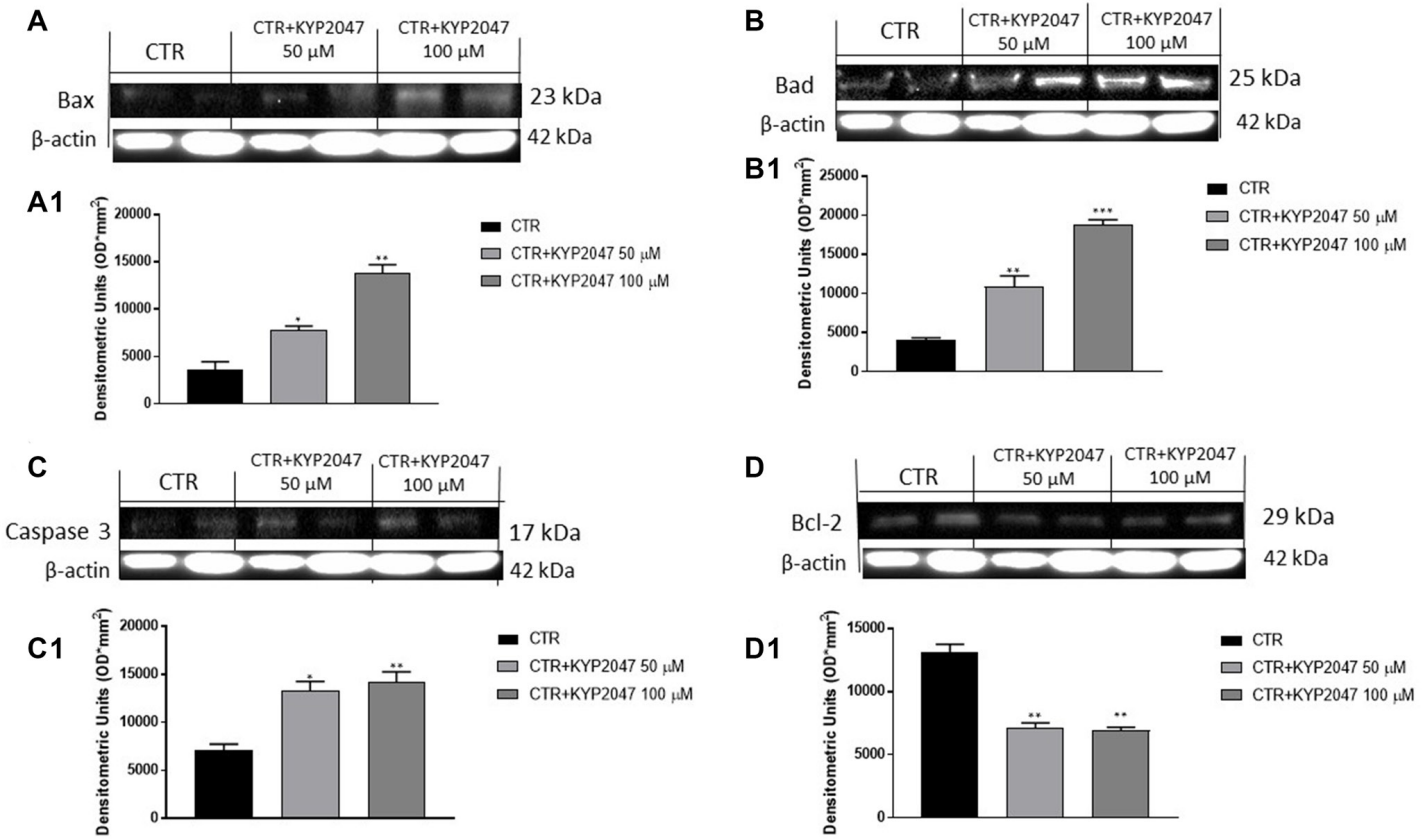
Effect of KYP-2047 on Bax, Bad, Caspase3 and Bcl-2 expression in CAL27 cells. The blots revealed an increase of pro-apoptotic Bax, Bad and caspase-3 expression and a decrease of Bcl2 expression following KYP-2047 treatment at concentrations of 50 μM and 100 μM compared to control group (**A**, **A1**, **B**, **B1**, **C**, **C1** and **D**, **D1**). Data are representative of at least three independent experiments. (A) ^*^ *p* < 0.05 vs. CTR; ^**^ *p* < 0.01 vs. CTR. (B) ^**^ *p* < 0.01 vs. CTR; ^***^ *p* < 0.001 vs. CTR; (C) ^*^ *p* < 0.05 vs. CTR; ^**^ *p* < 0.01 vs. CTR. (D) ^**^ *p* < 0.01 vs. CTR.

Our results showed that KYP-2047 at higher concentrations enhanced pro-apoptotic Bax, Bad and Caspase3 protein levels (Figure 2A, 2A1; 2B, 2B1; 2C, 2C1, respectively); whereas anti-apoptotic Bcl2 protein expression was significantly reduced following KYP-2047 treatment (Figure 2D, 2D1).

**Figure 3:**
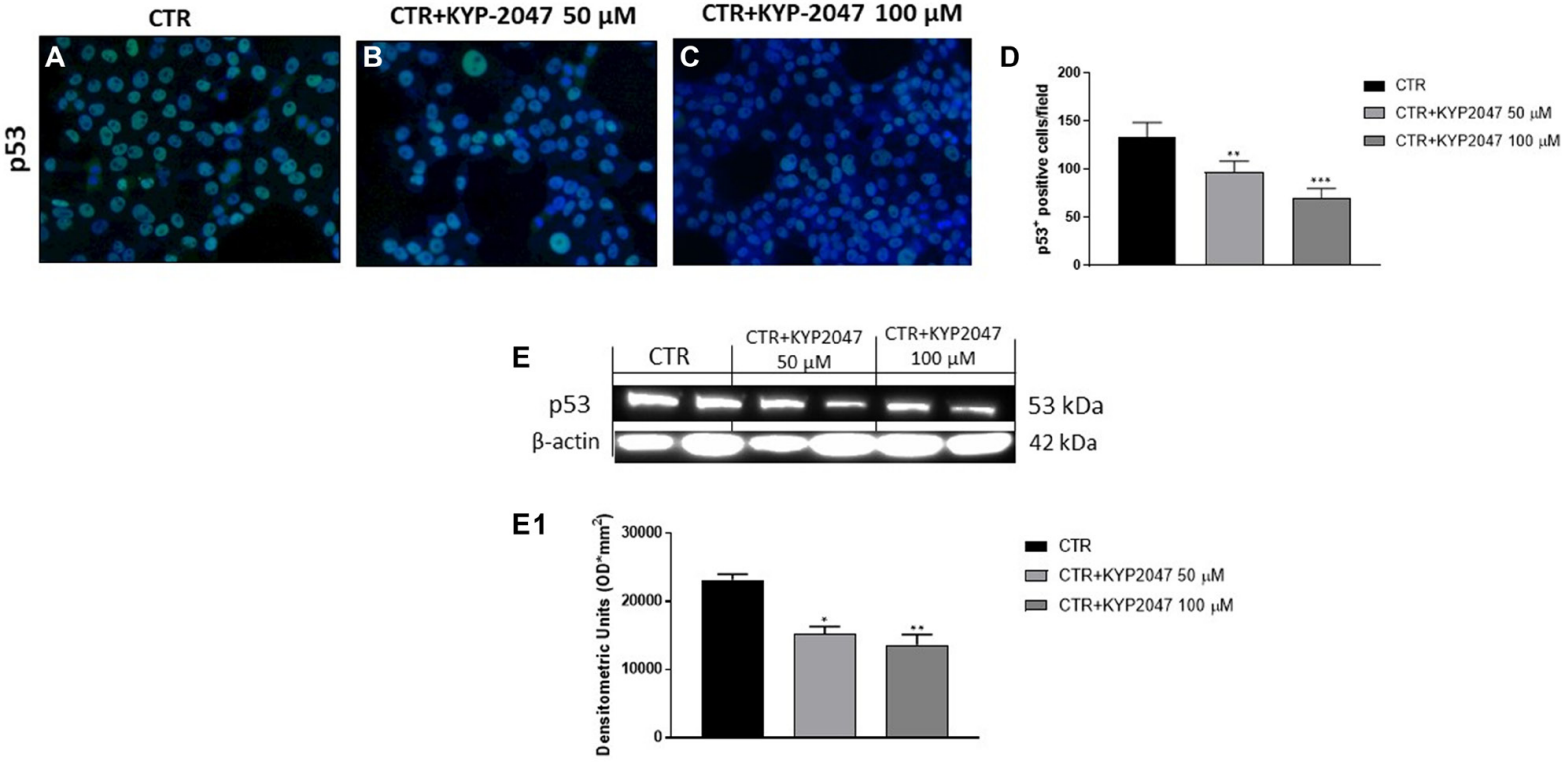
Effect of KYP-2047 on p53 expression in CAL27 cells. Immunofluorescence staining revealed a decrease of p53 expression following KYP-2047 treatment at concentrations of 50 μM and 100 μM compared to control group in a concentration-dependent manner (**A**–**C**). The data was confirmed also by western blot analysis (**E**, **E1**). Sections were observed and photographed at 40× magnification. Data are representative of at least three independent experiments. (**D**) ^**^ *p* < 0.01 vs. CTR; ^***^ *p* < 0.001 vs. CTR. (E, E1) ^*^ *p* < 0.05 vs. CTR; ^**^ *p* < 0.01 vs. CTR.

Genomic imbalances including gross chromosomal alterations and specific gene aberrations play a key role in oral cancer pathogenesis [27]. Among the genetic alterations involved in OSCC development, many studies focused on the role of mutant p53 [28, 29]. It has been demonstrated that mutations in the p53 protein can alter its function as a tumor suppressor and confer novel oncogenic capacities, promoting cancer growth [28]. Therefore, we decided to investigate the controversial role of p53 on CAL27 cells. In this context, our results demonstrated by immunofluorescence staining and western blot analysis that the control group was characterized by a high p53 expression, while KYP-2047 treatment at higher concentrations significantly reduced its expression in CAL27 cells, contrasting its controversial outcome and tumor growth (Figure 3A–3D; Figure 3E, 3E1).

#### KYP-2047 reduced angiogenesis in CAL27 cells

**Figure 4:**
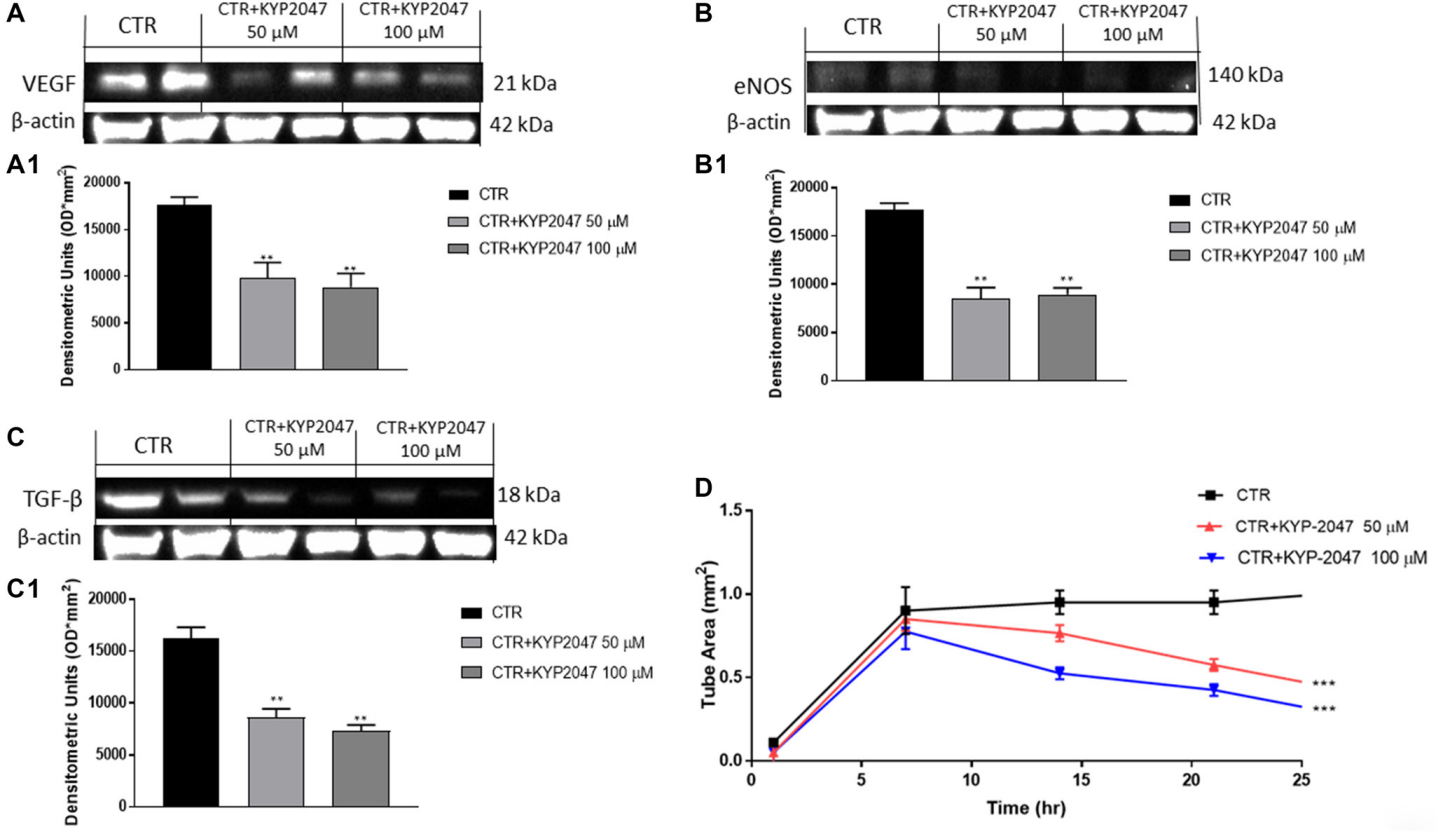
Effect of KYP-2047 on VEGF, eNOS and TGFβ expression in CAL27 cells. The blots showed a decrease of VEGF, eNOS and TGF-β expression following KYP-2047 treatment at concentrations of 50 μM and 100 μM compared to control group in a concentration-dependent manner (**A**, **A1**, **B**, **B1** and **C**, **C1**). Moreover, KYP-2047 treatment significantly reduced tubule network formation (**D**). Data are representative of at least three independent experiments. (A) ^**^ *p* < 0.01 vs. CTR. (B) ^**^ *p* < 0.01 vs. CTR. (C) ^**^ *p* < 0.01 vs. CTR. (D) ^***^ *p* < 0.001 vs. CTR.

Angiogenesis is a complex process that is essential for the growth of cancer [30]. Therefore, in this paper we detected angiogenesis factors expression as VEGF and eNOS. Our data revealed that KYP-2047 50 μM and 100 μM decreased VEGF and eNOS expression compared to control group (Figure 4A, 4A1 and 4B, 4B1).

Tumor growth is related to dysregulated transforming growth factor-β (TGF-β) expression [31]. TGF-β is secreted by various cell types residing in the tumor microenvironment (TME) and it has been demonstrated that TGF-β promotes tumor angiogenesis [31]. Our results showed that the treatment with KYP-2047 at higher concentrations significantly decreased TGF-β expression compared to control (Figure 4C, 4C1).

Moreover, to confirm the antiangiogenetic properties of KYP-2047 in CAL27 cells, we performed a Matrigel Tube Formation assay, showing that KYP-2047 50 μM and 100 μM significantly diminished tubule network formation (Figure 4D).

### *In vivo* studies

#### KYP-2047 reduced tumor growth

**Figure 5:**
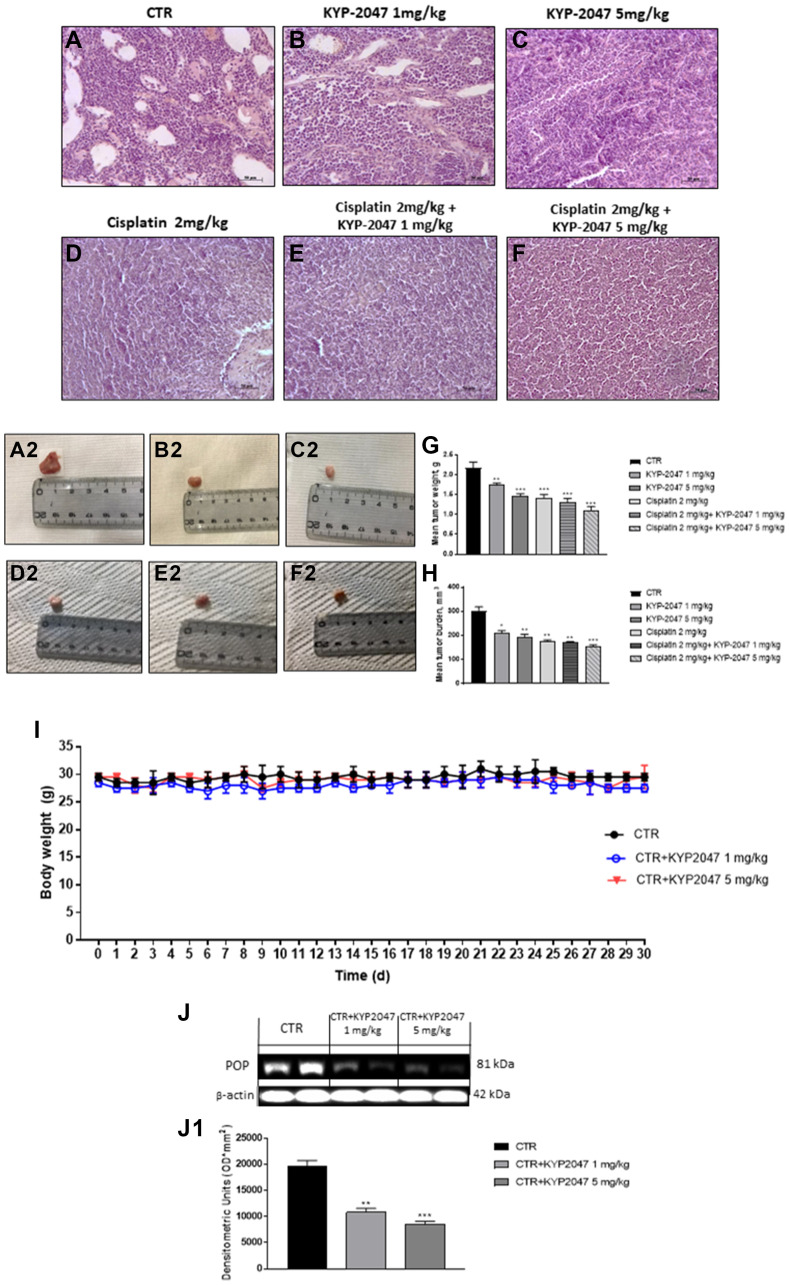
Effect of KYP-2047 on tumor growth. A significant tumor mass was observed in the control group (**A,** **A2**) whereas the treatment with KYP-2047 at doses of 1 mg/kg and 5 mg/kg significantly reduced tumor mass and neutrophil infiltration in a dose-dependent manner without encountering important animals body weight differences (**B**, **B2**, **C**, **C2**, **I**). The combinatory treatment between KYP-2047 and cisplatin significantly reduced tumor section much more than single components (**D, D2**, **E**, **E2** and **F**, **F2**), reducing also tumor weight as well as tumor burden (**G**, **H**). Furthermore, KYP-2047 treatment decreased POP expression (**J, J1**). Sections were observed and photographed at 20x magnification. Data are representative of at least three independent experiments. (G) ^**^ *p* < 0.01 vs. CTR; ^***^ *p* < 0.001 vs. CTR; (H) ^*^ *p* < 0.05 vs. CTR; ^**^ *p* < 0.01 vs. CTR; ^***^ *p* < 0.001 vs. CTR. (J, J1) ^**^ *p* < 0.01 vs. CTR; ^***^ *p* < 0.001 vs. CTR.

The histological evaluation of the control group (Figure 5A, 5A2) showed a marked subcutaneous tumor mass composed of vesicles, followed by an enhance of neutrophilic permeation; whereas KYP-2047 treatment 1 mg/kg and 5 mg/kg significantly decreased tumor sections and neutrophilic permeation in a dose-dependent manner (Figure 5B, 5B2 and 5C, 5C2). Moreover, based on these results, we decided to evaluate the combinatory treatment of KYP-2047 with the chemotherapy cisplatin on tumor tissues. Our results demonstrated that the combinatory treatment of cisplatin (2 mg/kg) and KYP-2047 (1 mg/kg and 5 mg/kg) significantly decreased tumoral sections much more than single components in a dose-dependent manner (Figure 5D, 5D2, 5E, 5E2, 5F and 5F2), inhibiting also tumor volume and weight as shown in the Figure 5G and 5H. During the course of treatment, no important change in animals’ weight was seen (Figure 5I).

In addition, to confirm the involvement of POP enzyme in tongue oral cancer progression, we investigated POP expression by western blot analysis, showing that KYP-2047 1 mg/kg and 5 mg/kg decreased its expression compared to control group in a dose-dependent manner (Figure 5J, 5J1).

#### KYP-2047 reduced mucosal lesions in CAL27-xenograft model

**Figure 6:**
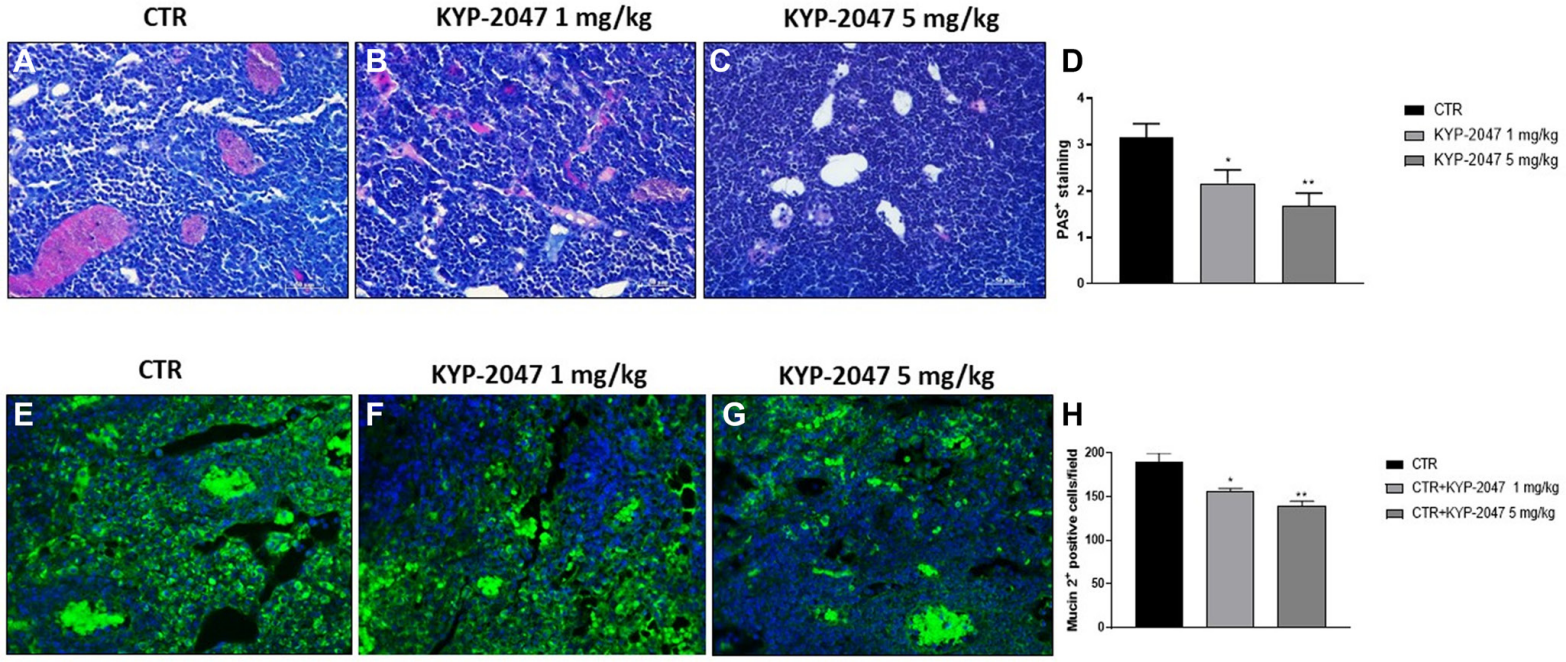
Effect of KYP-2047 on mucosal content. AB-PAS staining showed that the control group was characterized by a significant mucosal content, particularly high expression of acid and neutral mucins, whereas KYP-2047 at doses of 1 mg/kg and 5 mg/kg significantly reduced mucosal content (**A**–**C**). Moreover, immunofluorescence staining revealed high expression of Mucin 2 in the control, while KYP-2047 at doses of 1 mg/kg and 5 mg/kg significantly reduced its expression in a dose-dependent manner (**E**–**G**). Sections were observed and photographed at 20x and 40x magnifications. Data are representative of at least three independent experiments. (**D**) ^*^ *p* < 0.05 vs. CTR; ^**^ *p* < 0.01 vs. CTR. (**H**) ^*^ *p* < 0.05 vs. CTR; ^**^ *p* < 0.01 vs. CTR.

CAL27-xenograft model is characterized by the formation of small vesicles with glandular-like structures and high mucins expression that came into being at the end of the third week after cells inoculation [22, 32]. Therefore, in this study we decided to evaluate mucosal content by AB-PAS staining. Our results showed a significant mucosal content with an increase of acid mucins and neutral mucins levels stained in deep blue and bright magenta in the control group; instead, KYP-2047 treatment 1 mg/kg and 5 mg/kg significantly diminished mucins content in a dose-dependent manner (Figure 6A–6C, see PAS-positive staining score Figure 6D).

An aberrant expression of mucins, in particular of Mucin 2, has been reported in oral squamous carcinoma suggesting a strong relation to the outcome of patients affected [32]. Therefore, we investigated Mucin 2 expression by immunofluorescence assay, showing that KYP-2047 treatment significantly decreased its expression compared to control group in a dose-dependent manner (Figure 6E–6G, see Mucin2 positive cells score Figure 6H).

#### KYP-2047 increased apoptosis in CAL27-xenograft model

**Figure 7:**
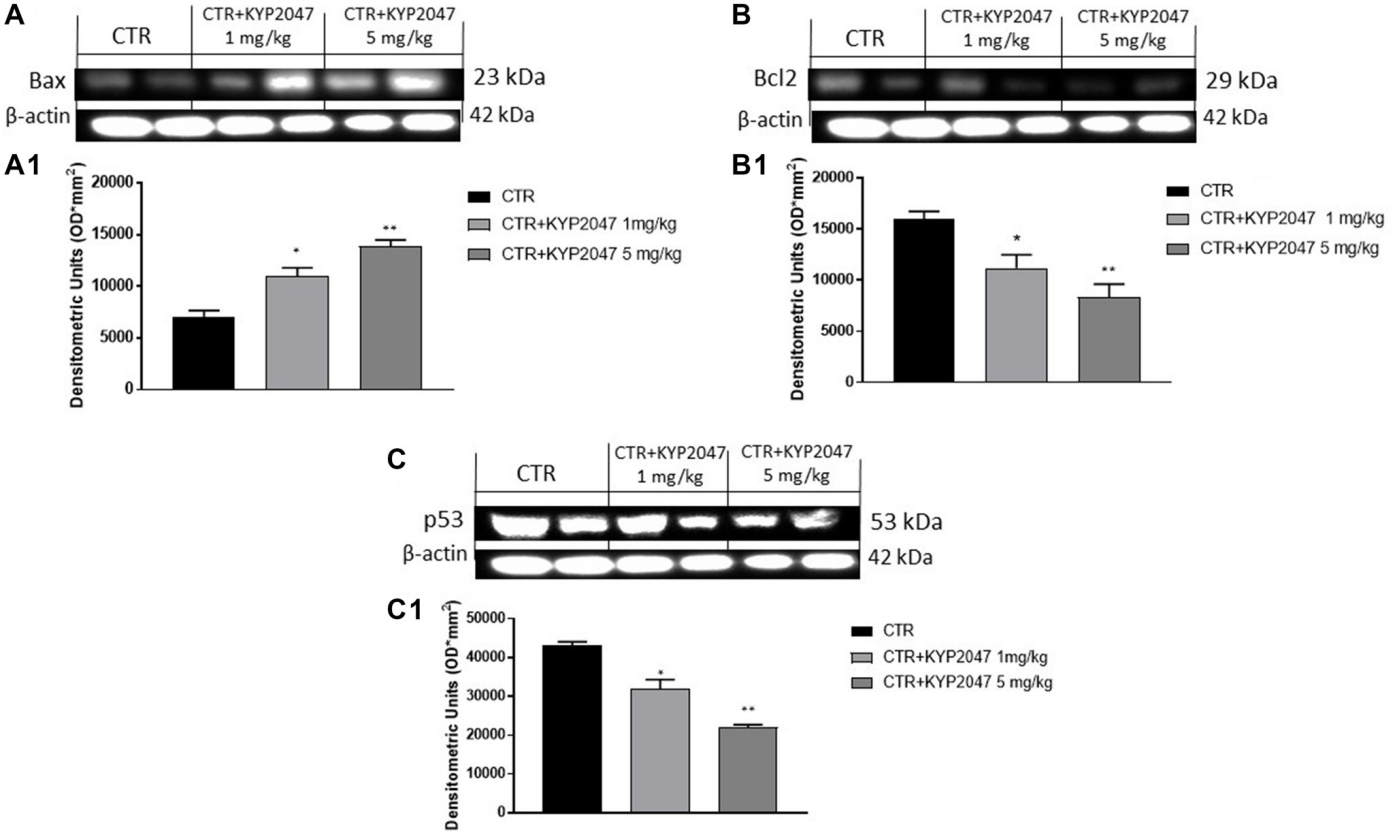
Effect of KYP-2047 on apoptosis pathway. The blots revealed an increase of pro-apoptotic Bax expression and a decrease of Bcl2 and p53 expression following KYP-2047 treatment at doses of 1 mg/kg and 5 mg/kg compared to control group in a dose-dependent manner (**A**, **A1**, **B**, **B1** and **C**, **C1**). Data are representative of at least three independent experiments. (A, A1) ^*^ *p* < 0.05 vs. CTR; ^**^ *p* < 0.01 vs. CTR. (B, B1) ^*^ *p* < 0.05 vs. CTR; ^**^ *p* < 0.01 vs. CTR. (C, C1) ^*^ *p* < 0.05 vs. CTR; ^**^ *p* < 0.01 vs. CTR.

Apoptosis alteration contributes to the progress of many pathologies such as oral cancer [33]. Therefore, we decided to investigate the ability of KYP-2047 to act on apoptosis process evaluating apoptotic markers. Our results revealed that KYP-2047 treatment 1 mg/kg and 5 mg/kg possess the ability to increase pro-apoptotic Bax expression (Figure 7A, 7A1), while Bcl2 expression was significantly reduced in a dose-dependent manner (Figure 7B, 7B1). Additionally, we examined p53 expression in oral cancer, showing that p53 expression was decreased following KYP-2047 treatment 1 mg/kg and 5 mg/kg compared to control group (Figure 7C, 7C1).

#### KYP-2047 reduced VEGF, eNOS and CD31 expression in CAL27-xenograft model

**Figure 8:**
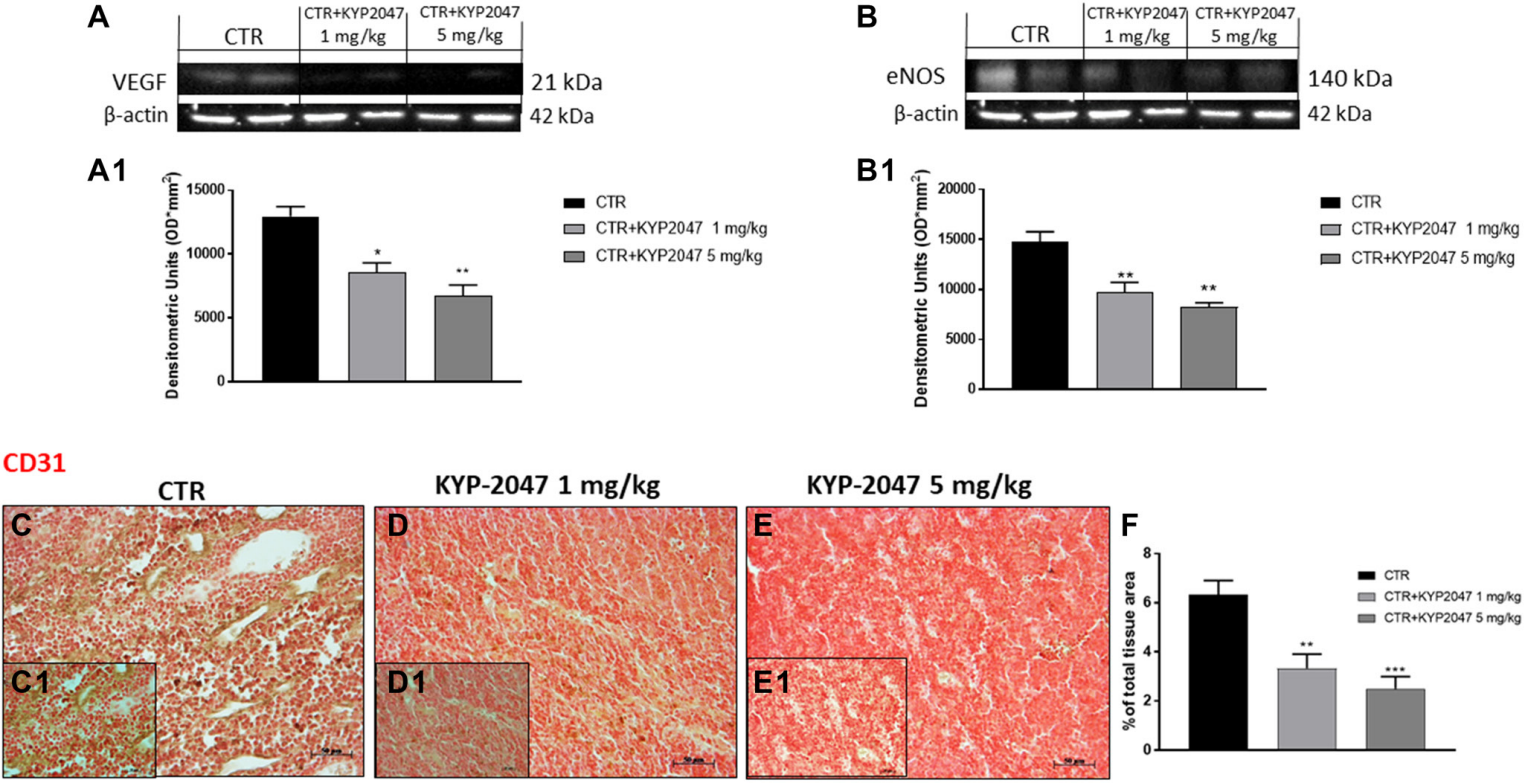
Effect of KYP-2047 on VEGF, eNOS and CD31 expression. The blots revealed a decrease of VEGF and eNOS expression following KYP-2047 treatment at doses of 1 mg/kg and 5 mg/kg compared to control group in a dose-dependent manner (**A**, **A1**, and **B**, **B1**). Immuhistochemical staining revealed that KYP-2047 treatment significantly reduced CD31 expression compared to control group (**C**, **C1**, **D**, **D1**, **E**, **E1**; see % of total tissue area score **F**). Data are representative of at least three independent experiments. (A) ^*^ *p* < 0.05 vs. CTR; ^**^ *p* < 0.01 vs. CTR. (B) ^**^ *p* < 0.01 vs. CTR. (F) ^**^ *p* < 0.01 vs. CTR; ^***^ *p* < 0.001 vs. CTR.

To confirm the *in vitro* results, we decided to investigate angiogenesis markers also in the xenograft model by evaluating VEGF and eNOS expression. Our data confirmed that KYP-2047 treatment 1 and 5 mg/kg possesses the ability to decrease VEGF and eNOS levels compared to control group in a dose-dependent manner (Figure 8A, 8A1 and 8B, 8B1 respectively).

Moreover, we evaluated the effect of KYP-2047 on CD31 expression, an important endothelial cell adhesion marker involved in oral cancer progression [34]. Immunohistochemical staining revealed that treatment with KYP-2047 decreased CD31 expression compared to control group (Figure 8C, 8C1, 8D, 8D1, 8E, 8E1, see % total of tissue area score 8F).

## DISCUSSION

Oral squamous cell carcinoma (OSCC) is a common cancer of the head and neck [35]. Among OSCC, tongue squamous cell carcinoma (TSCC) is enough widespread [36, 37]. The frequency of this cancer is growing in all world [37]. Several risk factors are strongly related to the development of this neoplasm, as smoking and excessive alcohol consumption [38]. TSCC is characterized by high rates of proliferation, recurrence and lymph node metastasis (LNM) [38]. TSCCs do not often have distant metastases, but recurrence of the cervical lymph nodes can occur in a large percentage of patients with TSCC, usually indicating a poor prognosis [39]. The analysis of risk factors for cervical lymph node recurrence is therefore essential to clarify the onset and progression of TSCC [39]. Despite a variety of novel approaches have been proposed for the treatment of OSCC, surgery remains the most effective strategy; however, the 5-year survival rate for oral cancer has not improved in the last decade [37, 40].

Currently, novel knowledge about specific regulatory pathways and signalling interactions that lead to neoplastic transformation and invasion has been gained [6]. Angiogenesis has a crucial role for TSCC growth [41, 42]. The formation of new blood vessels in tumor microenvironment contributes to tumor growth as well as tumor cell invasiveness [42]. Moreover, oral carcinogenesis has been linked to apoptosis process alteration [10]. It has been demonstrated that alterations of key regulatory apoptosis factors may lead to cancer, promoting metastasis, cell invasiveness and resistance to chemotherapy drugs [9, 10]. Thus, it is important to research novel molecular target against angiogenesis and/or apoptosis processes, to counteract TSCC progression. Recently, more attention was given to the role of Prolyl-oligopeptidase (POP) in cancer. POP is a serine protease constitutively expressed in all cells [14]. Recent studies revealed high levels of POP expression in many cancer types as glioblastoma, breast and gastric cancer [17–19], suggesting its involvement in tumor cell proliferation [17]. Therefore, in the last decade several POP inhibitors have been developed, as KYP-2047, to investigate their beneficial effect in many pathologies including cancer, showing surprising effects [19]. Thus, considering the keys role of apoptosis and angiogenesis in oral cancer and the involvement of POP enzyme in cancer pathogenesis, this paper aimed to investigate the beneficial effect of KYP-2047, a POP inhibitor, in an *in vitro* model of TSCC and in an *in vivo* xenograft model of oral cancer.

Primarily, we assessed the cytotoxicity of KYP-2047 in *a vitro* model of TSCC using CAL27, HSC-2 and HSC-3 cell lines. Our results demonstrated clearly that KYP-2047 significantly decreased cell viability in all three cell cultures at the same way.

Previous studies revealed that apoptosis process influences TSCC progression [43, 44]. Apoptosis mechanism is complex and involves many pathways [45]. A dysregulated apoptosis may contribute to cancer progression, promoting metastasis and cell invasiveness [45]. Thus, in this paper we detected pro-apoptotic markers as Bax, Bad and Caspase 3 and anti-apoptotic marker as Bcl2 in CAL27 cell lysates. Our data revealed that KYP-2047 significantly augmented Bax, Bad and Caspase-3 expression in a concentration-dependent manner; while anti-apoptotic Bcl2 expression was significantly reduced.

Oral squamous carcinoma is characterized by various genomic disparities, such as chromosomal alterations and gene aberrations [46]. Oncogene amplifications, mutations and suppressor gene deletions are responsible for the progressive development of malignant squamous epithelia [46]. Among genetic alterations that are involved in rise and progression of oral cancer, p53 gene alteration seems to be a very important event correlating with survival and with response rates to specific chemotherapy regimens [27, 29]. Mutations in the p53 protein can alter its tumor suppressor function, conferring new oncogenic features, and promoting tumor growth [29]. Therefore, we decided to investigate p53 expression in CAL27 cells which are characterized by mutant p53. In this context, the data revealed that control group was characterized by a high expression of p53; whereas KYP-2047 treatment significantly decreased its levels in a concentration-dependent manner, contrasting its controversial effect and tumor growth.

Moreover, angiogenesis plays a key role in TSCC progression [11]. Angiogenesis is an indispensable process for the progression of oral cancer due to the construction of novel blood vessels [11]. Thus, in this paper we detected angiogenic factors as VEGF and eNOS which regulate vascular homeostasis and vessel integrity [11]. In this context, our results demonstrated that KYP-2047 possesses the ability to significantly decrease VEGF and eNOS levels in a concentration-dependent manner. Additionally, KYP-2047 significantly reduced tubule network formation in a concentration-dependent manner, confirming its antiangiogenetic effect.

It has been demonstrated that cancer progression is related to dysregulated TGF-β expression [47]. TGF-β is a polypeptide factor which modulates apoptosis, cell differentiation and angiogenesis [47]. Various evidences revealed that TGF-β promotes cancer progression [48] including oral cancer [31, 47]. Therefore, in this paper we investigated TGF-β expression; our results showed that KYP-2047 treatment significantly decreased its level compared to control.

Following the auspicious data obtained by an *in vitro* model of TSCC, we decided to perform an *in vivo* xenograft model to further prove the abilities of KYP-2047. According to the *in vitro* data, the *in vivo* results revealed that KYP-2047 1 mg/kg and 5 mg/kg significantly reduced subcutaneous malignant mass and neutrophil infiltration compared to control, without encountering important animals weight differences. However, the combinatory treatment between KYP-2047 and the chemotherapy cisplatin significantly reduced tumoral sections much more than individual treatments in a dose-dependent manner, reducing also tumor volume. Moreover, KYP-2047 treatment significantly decreased POP levels in a dose-dependent manner, confirming its involvement in tongue oral cancer progression.

Oral squamous carcinoma is characterized by the formation of small vesicles accompanied by high content of mucins [49]. Mucins are high-molecular-weight glycoproteins implicated in various biological functions [32]; their aberrant expression has been reported in a variety of carcinomas [32]. Thus, we detected mucins content by AB-PAS staining, showing that the control group was characterized by a significant mucosal content, in particular high expression of acid and neutral mucins, while KYP-2047 treatment significantly reduced mucosal content.

In head and/or neck carcinomas, mucins expression, in particular Mucin 2, is often dysregulated [32]. It has been demonstrated that Mucin 2 contributes to neoplastic transformation, tumor survival, angiogenesis, and metastasis [32, 50]. Therefore, we decided to investigate Mucin 2 expression; in this context, our results showed that KYP-2047 treatment significantly reduced its appearance in a dose-dependent manner.

Furthermore, in this paper we evaluated apoptotic markers as Bax, Bcl2 and p53 in the xenograft model to prove the data previously obtained in the *in vitro* model, confirming that KYP-2047 treatment was able to significantly increase Bax expression and reduce Bcl2 and p53 expression. Additionally, we investigated angiogenesis markers in the xenograft model by evaluating VEGF, eNOS and CD31, a relevant endothelial cell adhesion marker, confirming that KYP-2047 treatment significantly reduced their expression in a dose-dependent manner.

Therefore, our data demonstrated the beneficial effect of KYP-2047 for OSCC treatment, proposing that it could be an alternative therapeutic strategy to counteract oral cancer progression. However, further investigations into the involvement of KYP-2047 in oral carcinogenesis will be required to fully understand its advantageous effect.

## MATERIALS AND METHODS

### *In vitro* studies

#### Cell cultures

Human oral tongue squamous carcinoma cell lines CAL27, HSC-2 and HSC-3 were purchased from ATCC (American Type Culture Collection, Rockville, MD, USA). CAL27 cells were cultured in Dulbecco’s Modified Eagle Medium (DMEM) (Life Technologies, Gibco^®^; Carlsbad, CA, USA) supplemented with 10% fetal bovine serum (FBS, Life Technologies, Gibco^®^; Carlsbad, CA, USA), 100 U/ml penicillin, and 100 μg/ml streptomycin. HSC-2 and HSC-3 cells were cultured in Minimum Essential Eagle’s Medium (Sigma-Aldrich) with 10% of fetal bovine serum (FBS) (Cultilab^®^, Campinas, Brazil), 100 U/mL penicillin and 100 μg/mL streptomycin (Sigma-Aldrich, St. Louis, MO, USA) in incubators under 5% CO_2_ at 37°C.

#### Cell treatment

CAL27, HSC-2 and HSC-3 cells were plated on 96-well plates at a density of 4 × 10^4^ cells/well to a final volume of 150 μl. After 24 hrs, CAL27, HSC-2 and HSC-3 cells were treated with KYP-2047 (Sigma-Aldrich^®^) for 24 hrs at increasing concentrations 0.01 μM, 0.1 μM, 1 μM, 10 μM, 30 μM, 50 μM and 100 μM dissolved in PBS.

#### Cell viability

Cell viability of CAL27, HSC-2 and HSC-3 cells was evaluated using a colorimetric assay for live cells (tetrazolium dye; MTT). KYP-2047 was tested in all three cell cultures at different concentrations for 24 hrs and then incubated at 37°C with MTT (0.2 mg/mL) for 1 h, as previously described [51].

Experimental groups:

1. Control group: TSCC cell lines CAL27, HSC-2 and HSC-3;
2. KYP-2047 0.01 μM group: CAL27, HSC-2 and HSC-3 cells were treated with KYP-2047 0.01 μM for 24 hrs;
3. KYP-2047 0.1 μM group: CAL27, HSC-2 and HSC-3 cells were treated with KYP-2047 0.1 μM for 24 hrs;
4. KYP-2047 1 μM group: CAL27, HSC-2 and HSC-3 cells were treated with KYP-2047 1 μM for 24 hrs;
5. KYP-2047 10 μM group: CAL27, HSC-2 and HSC-3 cells were treated with KYP-2047 10 μM for 24 hrs;
6. KYP-2047 30 μM group: CAL27, HSC-2 and HSC-3 cells were treated with KYP-2047 30 μM for 24 hrs;
7. KYP-2047 50 μM group: CAL27, HSC-2 and HSC-3 cells were treated with KYP-2047 50 μM for 24 hrs;
8. KYP-2047 100 μM group: CAL27, HSC-2 and HSC-3 cells were treated with KYP-2047 100 μM for 24 hrs;

For other analysis we continued to analyze only KYP-2047 50 μM and 100 μM because represented the most cytotoxic concentrations revealed by MTT assay. Moreover, since KYP-2047 showed similar effects on cell viability in all three cell lines, we decided to continue to analyze the effect of KYP-2047 only on CAL27 cell line because it represents one of the most frequently used cell line in the field of OSCC [22, 23].

#### Western blot analysis

Western Blot analysis on CAL27 cell culture was performed as previously described [52]. The following primary antibodies were detected: anti-Bax (1:500; Santa Cruz Biotechnology, Dallas, TX, USA; sc-7480); anti-Bcl2 (1:500; Santa Cruz Biotechnology, Dallas, TX, USA; sc-7382), anti-p53 (1:500; Santa Cruz Biotechnology, Dallas, TX, USA; sc-126); anti-Bad (1:500, Santa Cruz Biotechnology, Dallas, TX, USA; sc), anti-caspase3 (1:500, Santa Cruz Biotechnology, Dallas, TX, USA; sc-56053), anti-vascular endothelial growth factor (VEGF) (1:500; Santa Cruz Biotechnology, Dallas, TX, USA; sc-7269); anti-endothelial nitric oxide synthase (eNOS) (1:500; Santa Cruz Bio-technology, Dallas, TX, USA; sc-376751); anti-transforming growth factor beta (TGFβ) (1:500, Santa Cruz Biotechnology, Dallas, TX, USA; sc-130348); anti-βactin for cytosolic fraction (1:500; Santa Cruz Biotechnology; Dallas, TX, USA. sc-8432) and anti-lamin A/C for nuclear fraction (1:500; Santa Cruz Biotechnology; Dallas, TX, USA, sc-376248). Signals are perceived with enhanced chemiluminescence (ECL) detection system mixture (Thermo Fisher, Waltham, MA, USA).

#### Immunofluorescence assay

Immunofluorescence assay on CAL27 cell culture was executed as discussed by Donaldson [53]. The following primary antibody anti-p53 (1:500; Santa Cruz Biotechnology, Dallas, TX, USA; sc-126) was used. Sections were observed and photographed using a Leica DM2000 microscope (Leica).

#### Matrigel tube formation assay

Tube assays formation was executed using a minimal volume of Matrigel (0.24 mg/cm^2^) as described previously [54]. Tube area quantification was performed using Metamorph software (Universal Imaging Corporation, West Chester, PA) as described [54].

### *In vivo* studies

#### Animals

BALB/c nude male mice were obtained from Jackson Laboratory (Bar Harbor, Hancock, ME, USA). Animals were fed with a standard diet and water *ad libitum* under pathogen-free conditions with a 12 h light/12 h dark. Animal study was approved by the University of Messina (n◦ 368/2019-PR released on 14 May 2019) in accordance with Italian regulations on the use of animals (D.M.116192) and Council Regulation regulations (EEC) (O.J. of E.C. L 358/1 12/18/1986).

#### Experimental design

Xenograft tumor model was performed by inoculating subcutaneously of 3 × 10^6^ CAL27 cells per tumor in 0.2 mL of Phosphate Buffered Saline (PBS) and 0.1 mL Matrigel (BD Bioscience, Bedford, MA) as discussed by Zhu [55]. After tumor cell inoculation, mice were monitored daily for body weight, morbidity and mortality. After 1 week of tumor induction, mice were separated randomly into 6 groups. Mice were treated with KYP-2047 1 mg/kg and 5 mg/kg every three days according to bibliography [19, 20, 56]. It was solubilized in PBS with 0.001% of dimethyl sulfoxide. Moreover, we decided to evaluate the combinatory treatment of KYP-2047 with cisplatin chemotherapy on tumor tissue. The dose of cisplatin (2 mg/kg per body weight, dissolved in 200 μL of sterilized water) was chosen according to bibliography [57, 58]. The tumor size was estimated using a caliper and calculated as: V = W^2^ × L/2, where W and L were the minor and major length. After 30 days from tumor cell inoculation, animals were sacrificed and tumours were excised to perform several assays.

Mice were separated into 6 groups:

1. Control group: weekly intravenous (iv) administration of saline.
2. Control group+ KYP-2047 1 mg/kg: intraperitoneal (ip) administration of KYP-2047 (1 mg/kg dissolved in PBS) every three days until 30 days.
3. Control group+ KYP-2047 5 mg/kg: intraperitoneal (ip) administration of KYP-2047 (5 mg/kg dissolved in PBS) every three days until 30 days.
4. Control group+ Cisplatin 2 mg/kg: intraperitoneal (ip) administration of Cisplatin (2 mg/kg dissolved in sterilized water) every three days until 30 days.
5. Control group+ Cisplatin 2 mg/kg + KYP-2047 1 mg/kg: intraperitoneal (ip) administration of Cisplatin (2 mg/kg dissolved in sterilized water) and KYP-2047 (1 mg/kg dissolved in PBS) every three days until 30 days.
6. Control group+ Cisplatin 2 mg/kg + KYP-2047 5 mg/kg: intraperitoneal (ip) administration of Cisplatin (2 mg/kg dissolved in sterilized water) and KYP-2047 (5 mg/kg dissolved in PBS) every three days until 30 days.

#### Haematoxylin and eosin (H&E) staining

H&E assay was executed as before pronounced [52]. Tumor samples were deparaffinized with xylene and stained with H&E staining. The images were shown at 20x magnification (50 μm of the Bar scale) using an Axiovision Zeiss microscope (Milan, Italy).

#### Alcian Blue and Periodic Acid–Schiff (AB-PAS) staining

To evaluate mucosal content in the tumor tissues, paraffin-embedded sections were stained with Alcian Blue-Periodic Acid-Schiff (AB-PAS) as previously described [59] according to the manufacturer’s instructions (Bio-optica, Italy). The Alcian blue solution at a pH of 2.5 stained all acid mucins deep blue; while the subsequent application of the PAS technique stained the neutral mucins bright magenta. All sections were analysed by a histopathologist using an Axiovision Zeiss microscope (Milan, Italy). The images were photographed at 20x magnification (50 μm of the Bar scale).

#### Western blot analysis

Western Blot analysis on tumor samples was performed as previously described [60]. The membranes were incubated with the primary antibodies: anti-Bax (1:500; Santa Cruz Biotechnology, Dallas, TX, USA; sc-7480); anti-Bcl2 (1:500; Santa Cruz Biotechnology, Dallas, TX, USA; sc-7382), anti-p53 (1:500; Santa Cruz Biotechnology, Dallas, TX, USA; sc-126); anti-vascular endothelial growth factor (VEGF) (1:500; Santa Cruz Biotechnology, Dallas, TX, USA; sc-7269); anti-endothelial nitric oxide synthase (eNOS) (1:500; Santa Cruz Bio-technology, Dallas, TX, USA; sc-376751); anti-POP (1:500; Santa Cruz Biotechnology, Dallas, TX, USA; sc-365416); anti-βactin for cytosolic fraction (1:500; Santa Cruz Biotechnology; Dallas, TX, USA. sc-8432) and anti-lamin A/C for nuclear fraction (1:500; Santa Cruz Biotechnology; Dallas, TX, USA, sc-376248). Signals were perceived with enhanced chemiluminescence (ECL) detection system mixture according to the manufacturer’s instructions (Thermo Fisher, Waltham, MA, USA).

#### Immunofluorescence assay

Immunofluorescence assay was executed as pronounced [61]. Tissue sections of 7 μm were incubated with the following primary antibody anti-Mucin 2 at 37°C overnight (1:100; Santa Cruz Biotechnology, Dallas, TX, USA). After the incubation with the primary antibody, the sections were washed with PBS and incubated with a secondary antibody Alexa Fluor 488 goat anti-mouse (1:1000 v/v Molecular Probes, UK) for 1 h at room temperature. 4′,6′-diamidino-2-phenylindole (DAPI; Hoechst, Frankfurt; Germany) 2 μg/ml in PBS was added for nuclear staining. The images were photographed at 40x magnification using an optical microscope (Zeiss, Axio Vision).

#### Immunohistochemistry assay

Immunohistochemical localization for CD31 antibody (1:100; Santa Cruz Biotechnology, Dallas, TX, sc- 376764) was made as before defined by Scuderi et al. [19]. The images were shown at a magnification of 20x and 40x (50 μm and 20 μm of the bar scale, respectively) using an optical microscope (Zeiss, Axio Vision, Feldbach, Schweiz).

#### Materials

KYP-2047 and other chemical reagents are purchased by Sigma-Aldrich (Milan, Italy).

#### Statistical evaluation

The data showed in the figures are illustrative of at minimum 3 experiments executed on different experimental days. The data were examined using one-way ANOVA analysis followed by a Bonferroni post-hoc test for multiple comparisons. It was considered significant a *p*-value of less than 0.05.

## CONCLUSIONS

In conclusion, the results obtained demonstrated that KYP-2047 treatment was able to modulate angiogenesis and apoptosis processes, offering new insight into their roles in oral cancer pathogenesis. Therefore, KYP-2047, a POP inhibitor, could be a possible therapeutic strategy to contrast oral cancer growth through angiogenesis and apoptosis modulation.

## Abbreviations

OSCC: Oral squamous cell carcinoma
TSCC: Tongue squamous cell carcinoma
POP: Prolyl oligopeptidase
PREP: Prolyl endopeptidase
KYP-2047: 4-phenyl-butanoyl-l-prolyl-2(S)-cyanopyrrolidine
eNOS: Endothelial nitric oxide synthase
TGF-β: Transforming Growth Factor β
VEGF: Vascular endothelial growth factor
